# A Rare Case of Granulomatosis With Polyangiitis (Wegener’s Granulomatosis) Presenting With Otologic Symptoms and Facial Paralysis

**DOI:** 10.7759/cureus.74602

**Published:** 2024-11-27

**Authors:** Lucas Kim, Mobeen Shirazi

**Affiliations:** 1 Otolaryngology - Head and Neck Surgery, Affiliated Ear Nose and Throat Physicians, Woodstock, USA

**Keywords:** asymmetric facial paralysis, granulomatosis with polyangiitis (gpa), myringotomy, severe otalgia, wegener’s granulomatosis

## Abstract

Granulomatosis with polyangiitis (GPA) is an uncommon disorder that causes inflammation of the blood vessels in the nose, throat, lungs, and kidneys. We describe a 20-year-old female who presented to the emergency room with acute left facial paralysis, worsening left-sided otalgia secondary to a hyperemic tympanic membrane with middle ear granulation tissue, nasal obstruction with associated intermittent epistaxis, and cough. A high index of suspicion for GPA helped make an early diagnosis with subsequent initiation of treatment. This case details an unusual presenting symptom for GPA, as well as our treatment plan and the patient’s subsequent recovery.

## Introduction

Granulomatosis with polyangiitis (GPA), formerly known as Wegener's Granulomatosis, is an uncommon multi-system autoimmune disorder that has a multi-organ involvement and typically affects the respiratory tract and is known to also affect the sinuses and ears. This disorder induces necrotizing vasculitis and granulomatous inflammation throughout systemic vasculature and is characterized by necrotizing granulomas and pauci-immune vasculitis [1]. Facial paralysis is a rare presentation for GPA. In addition, the manifestation of GPA in the middle ear is uncommon. In this report, we describe a previously healthy 20-year-old female who presented to the emergency room with worsening left-sided otalgia and acute left facial paralysis. A high degree of suspicion and the patient's concurrent symptoms of cough, nasal obstruction, and epistaxis helped make an early diagnosis of GPA.

## Case presentation

**Figure 1:**
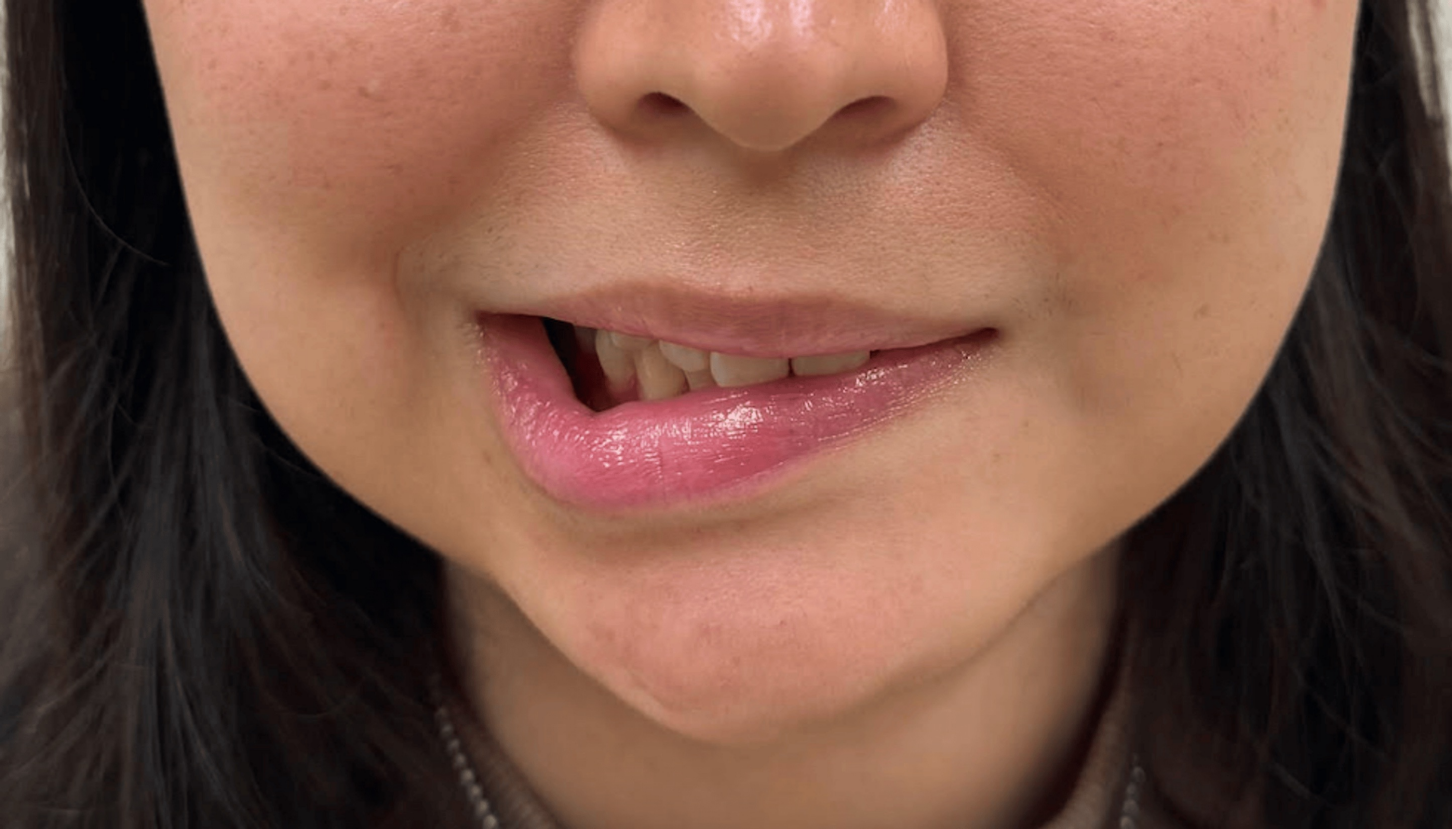
Acute left facial paralysis

A 20-year-old female presented to the emergency room with worsening left-sided otalgia and acute left-sided facial paralysis (Figure 1).

**Figure 2:**
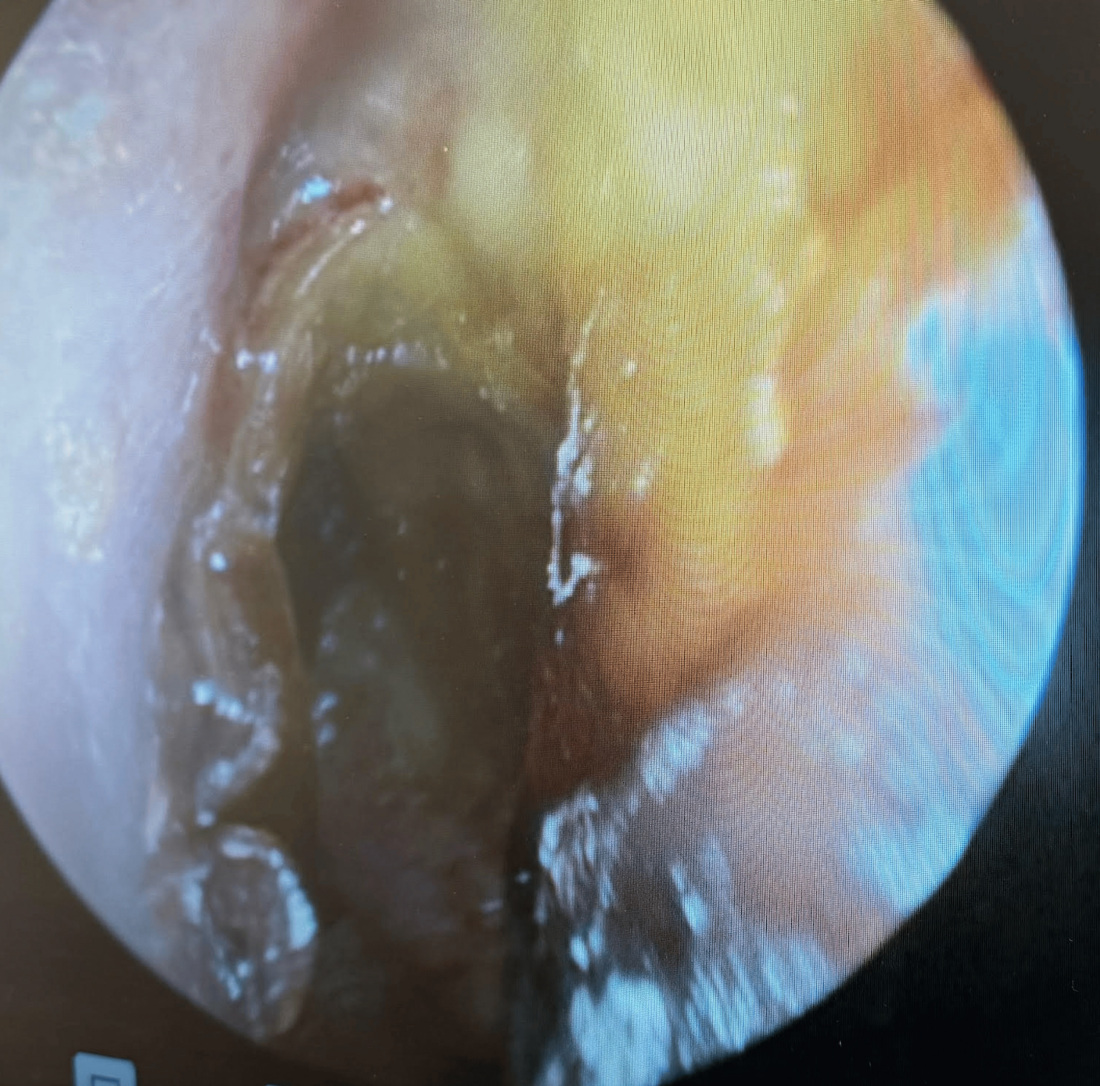
Inflamed nasal granulation tissue

**Figure 3:**
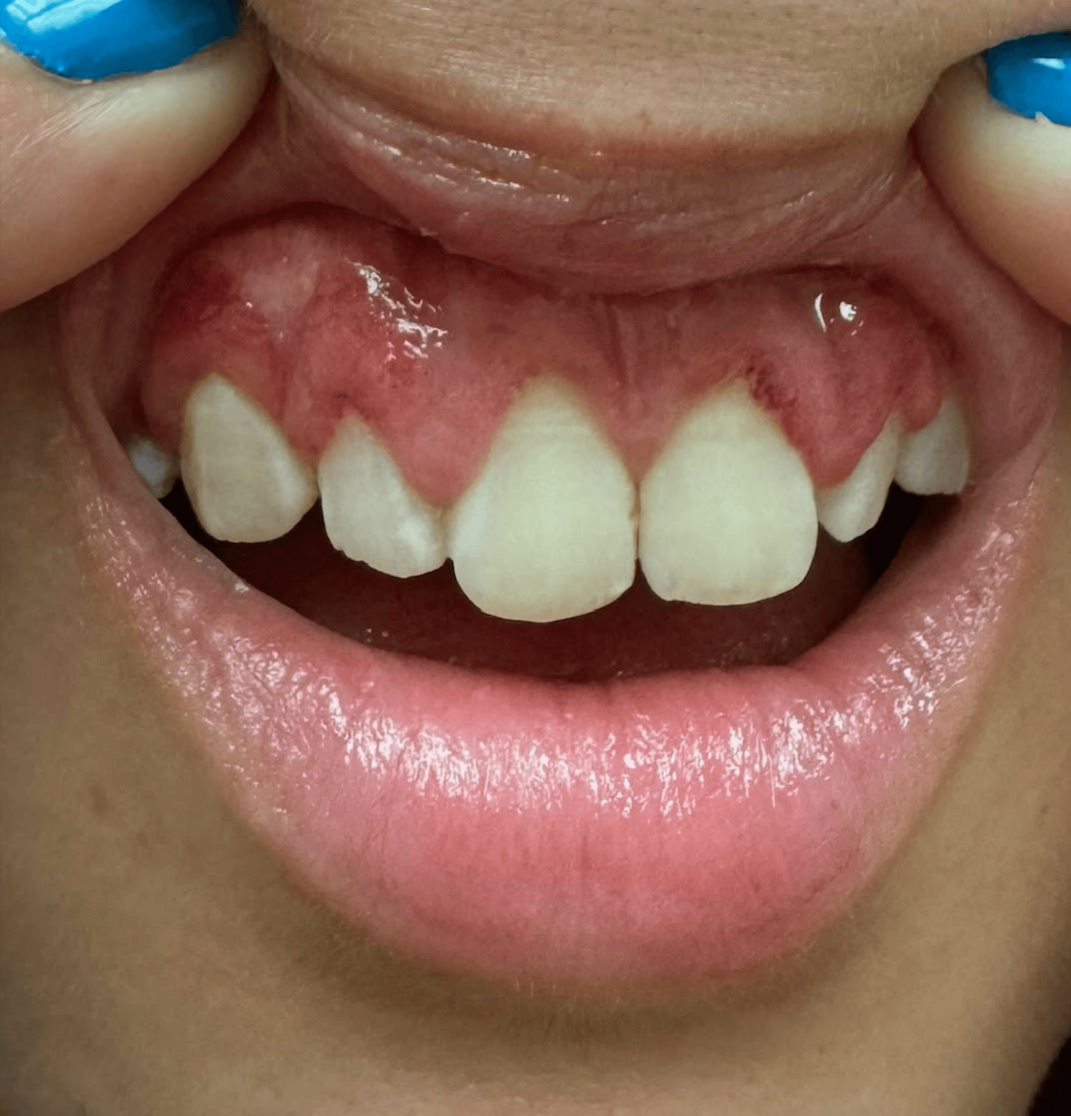
Inflamed gingival mucosa

The patient reported an approximately three-week history of fullness in the left ear with an associated cough. Her primary care physician had treated her with oral antibiotics and topical ear drops. Her otologic symptoms were worsening, and when she developed acute facial paralysis, she presented to the emergency room. She reported significant left aural fullness with associated otalgia, dry cough, epistaxis, and nasal obstruction. She denied any hemoptysis, dysphagia, or odynophagia. Physical examination revealed complete left facial paralysis such that the patient was unable to adequately close her left eye. Otologic examination revealed an inflamed left tympanic membrane. A nasal examination revealed significant bilateral crusting in the anterior nasal cavity (Figure 2). The intranasal tissue was found to be friable. An oral cavity exam revealed inflammatory changes in the patient’s gingival mucosa (Figure 3).

**Figure 4:**
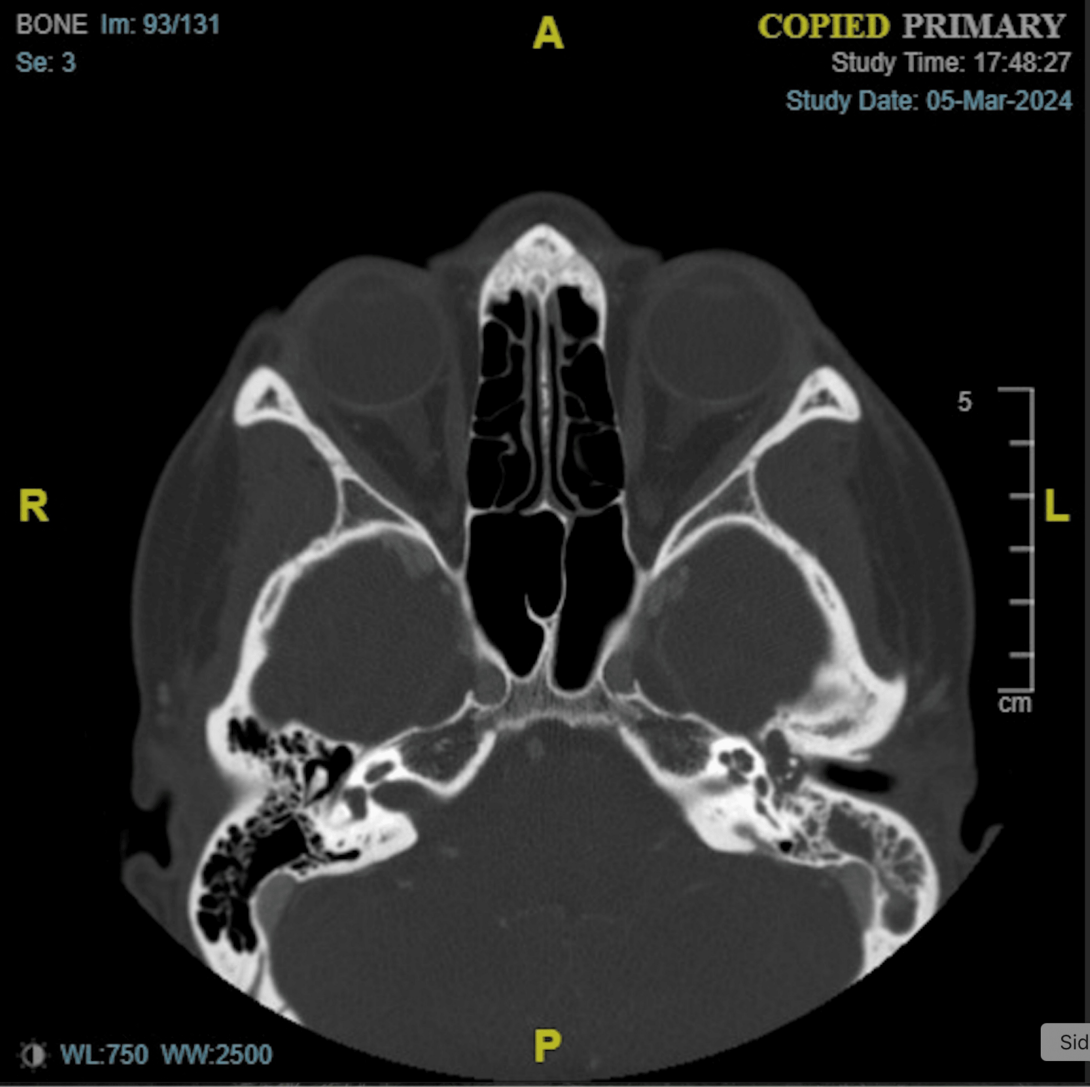
Opacification of the left middle ear and mastoid air cells, without bony erosion

**Figure 5:**
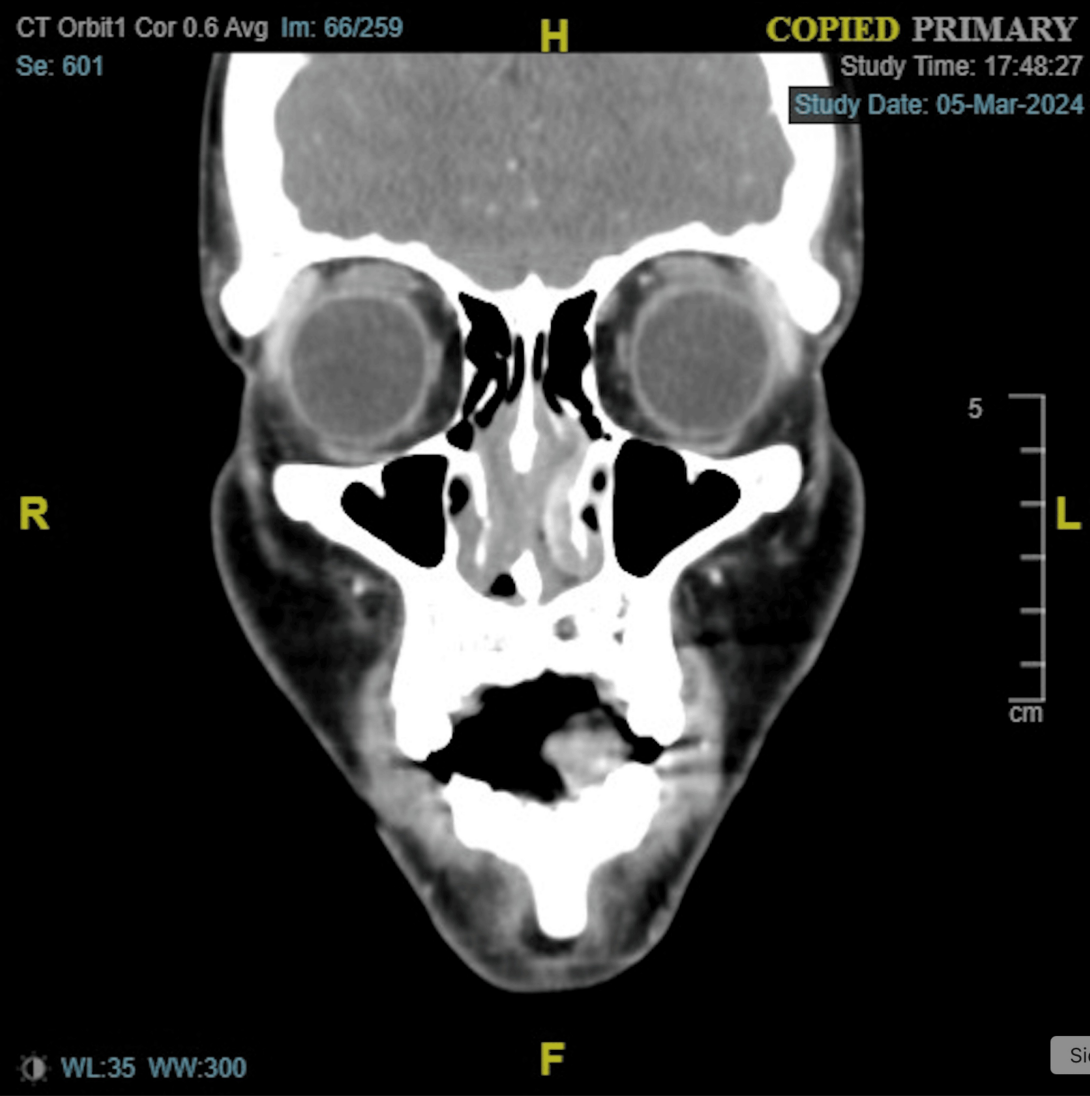
Anterior nasal airway soft tissue opacification with septal perforation

**Figure 6:**
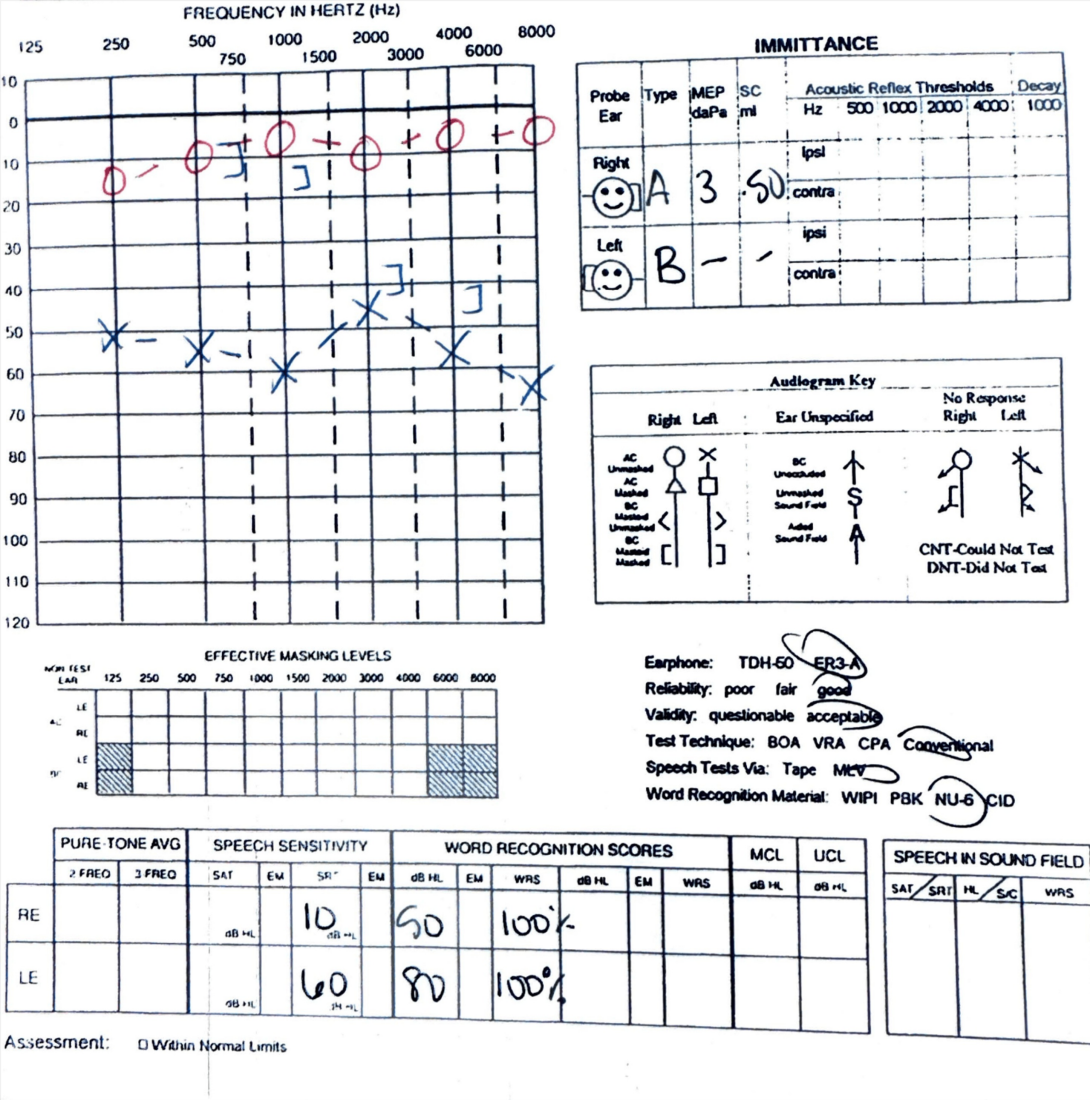
Audiogram demonstrating unilateral conductive hearing loss in left ear

The remainder of the head and neck exam was unremarkable. A contrast-enhanced CT of the face was performed in the emergency room. Opacification of the left middle ear and left mastoid air cells was present, but there was a lack of any bony erosion (Figure 4). Soft tissue opacification of the anterior nasal airway was observed, potentially indicating an underlying septal perforation (Figure 5). An audiogram revealed a unilateral conductive hearing loss in her left ear (Figure 6).

**Figure 7:**
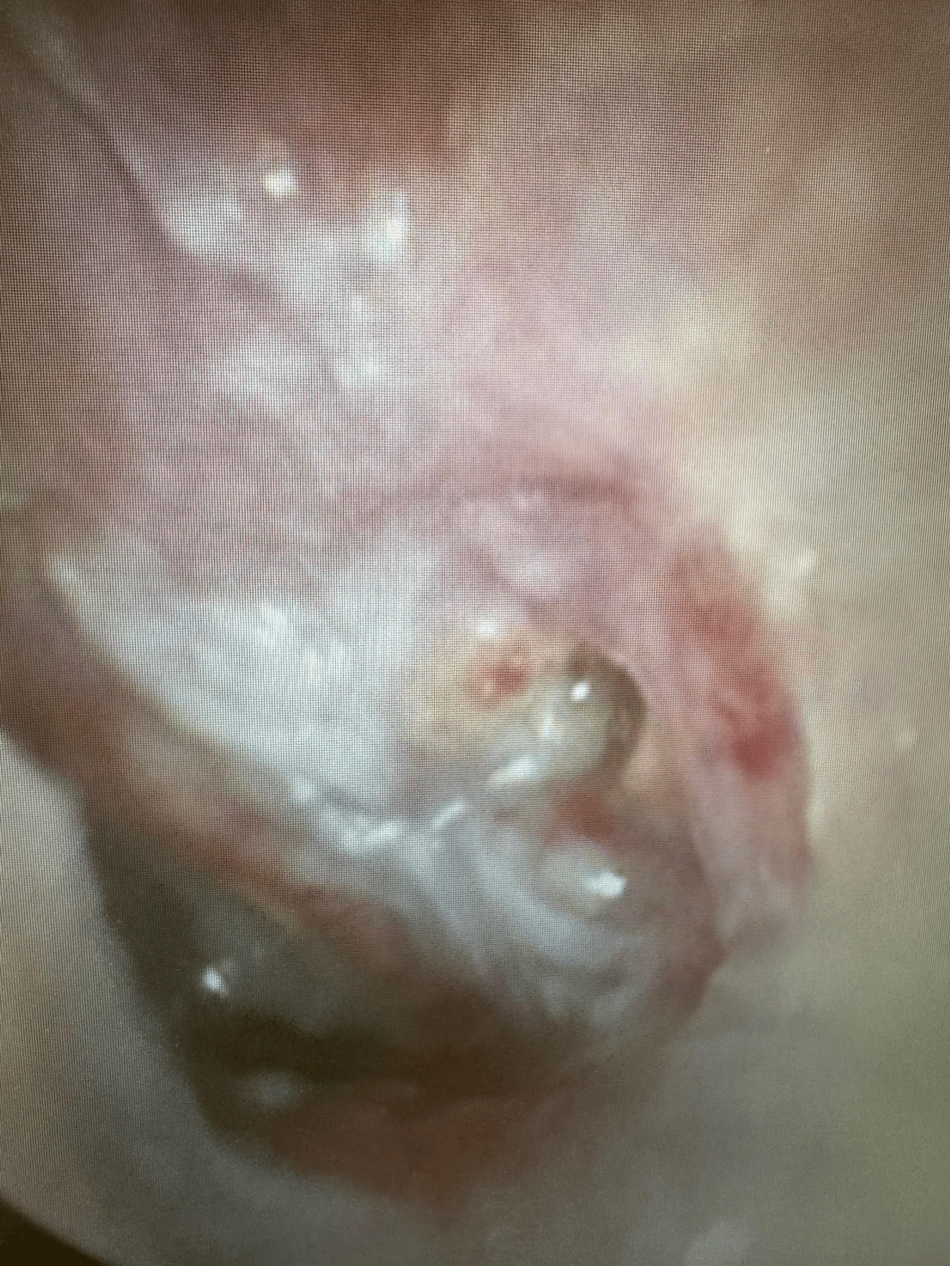
Inflamed and hyperemic left tympanic membrane

**Figure 8:**
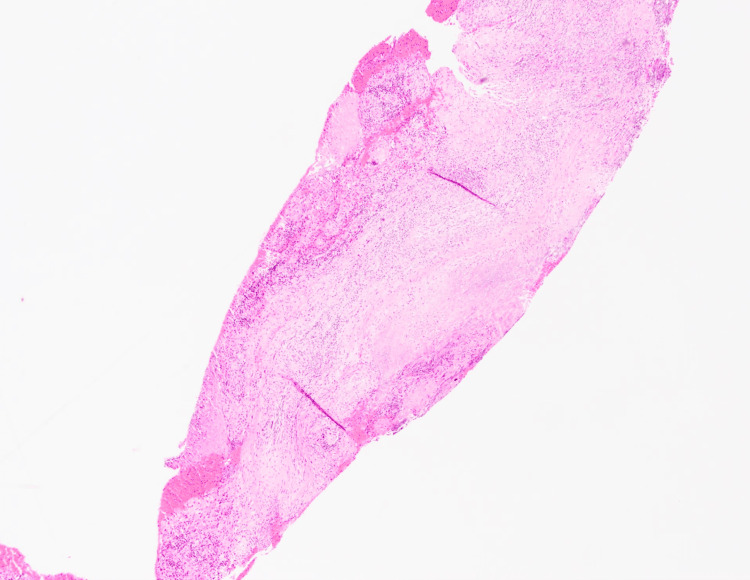
Intranasal tissue biopsy revealing acute vessel wall inflammation with underlying necrosis

**Figure 9:**
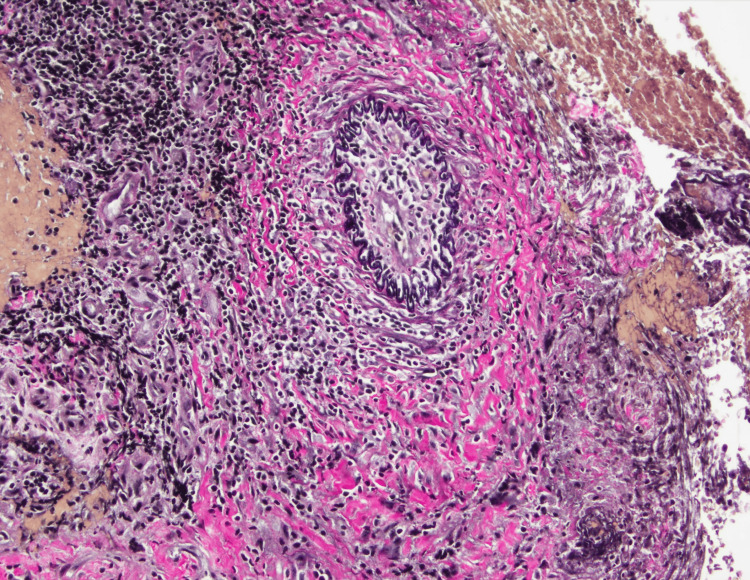
Elastic stain revealing the vasculitis in the vessel wall

Given this history and accompanying symptoms, the patient was taken to the operating room for a planned biopsy of the inflammatory anterior nasal tissue. A wide-left myringotomy was performed to help decompress the inflammatory micro-environment around the facial nerve. Examination of the left tympanic membrane revealed an inflamed, hyperemic tympanic membrane with underlying granulation tissue (Figure 7). A biopsy of the intranasal tissue revealed acute vessel wall inflammation with underlying necrosis (Figure 8). In addition, elastic stain results further highlighted the vasculitis in the vessel wall (Figure 9).

**Figure 10:**
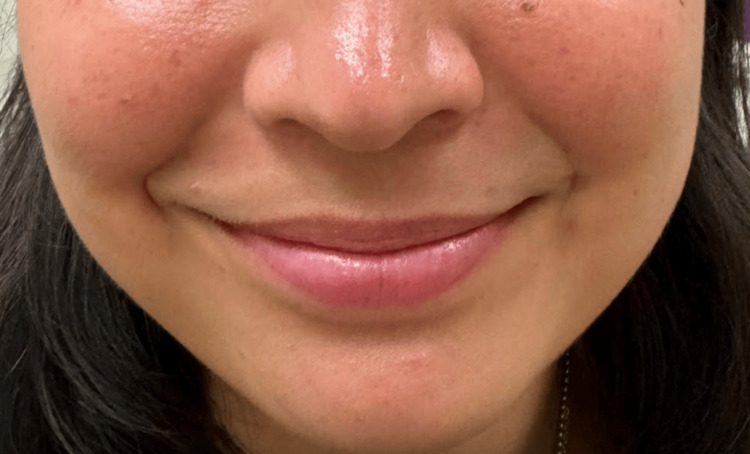
Complete recovery of left facial nerve function

Due to a high index of suspicion for underlying vasculitis, serological testing for pan cytoplasmic granular staining pattern cytoplasmic antineutrophil cytoplasmic antibodies (c-ANCA) was performed. c-ANCA was found to be highly elevated. The patient was started on IV steroids while in the hospital and subsequently transitioned to high-dose oral steroids. A subsequent chest X-ray and CT chest revealed no abnormal findings. After seeing a rheumatologist, the patient was kept on high-dose oral steroids and then started on Rituximab. Ophthalmology evaluation revealed no exposure keratitis in the left eye. Subsequent evaluation in the office after approximately two months of treatment revealed reduced crusting within the nasal cavity, a decrease in the inflammatory changes of the left tympanic membrane and middle ear, resolution of the patient’s gingival mucosal inflammation and cough, and improving left facial movement that permitted complete left eye closure. Approximately five months after initial presentations, the patient had normal facial nerve function (Figure 10).

## Discussion

GPA is a rare disease that has a reported annual incidence of 12.8 cases/million person-years in working-age adults in the US, over seven-fold higher than the annual incidence in children [2]. Studies of ethnicity as a risk factor have been generally inconclusive, though some results may indicate a higher prevalence in Caucasians [3,4]. 

GPA has been shown to often affect the respiratory tract, lungs, kidneys, and systemic vasculature. Inflammation of the gastrointestinal mucosa and oral gums has been noted in some cases [5], as autoimmune disorders often compromise the mucosal lining of multiple organ systems. As seen in this particular patient, facial nerves may be affected by GPA. Both bilateral and unilateral cases of facial palsy have been noted in published literature [6,7]. Such facial paralysis may result from infection in the facial nerve lining, leading to edema and nerve compression, preventing the conduction of neural signals. 

Patients with GPA most commonly present with chronic sinusitis, chronic otitis media, and epistaxis. Dysfunctions in the upper respiratory system are present in around 90% of all GPA patients [8]. Patients are also known to exhibit hematuria, joint pain, nephritis, conjunctivitis, and nasal ulcerations. Chronic inflammation of the nasal mucosa can lead to anterior crusting rhinitis and septal perforations. A notable avenue for a GPA diagnosis is via a c-ANCA test. This test screens for antineutrophil cytoplasmic antibodies (ANCA) in a patient’s blood. These antibodies attack neutrophils and may indicate the presence of an autoimmune disorder if their levels are abnormal. Patients suffering from GPA often exhibit positive results in c-ANCA tests, though around 10% of patients with GPA test negative [9]. Upon biopsy analysis, necrosis and granulomatous also serve as telltale symptoms of GPA. Though this patient initially presented with facial palsy, it is a rare symptom, having only a 5-8% incidence in patients presenting GPA [10]. Glucocorticoids and immunosuppressants are a standard and generally successful treatment for patients with GPA exhibiting facial palsy in regaining motor function [7,11]. Performing a right myringotomy upon initial presentation helped reduce some of the inflammation in the middle ear. A pressure equalization (PE) tube was not placed secondary to the granulation tissue already present in the middle ear, as we worried that placing a PE tube into the granulation tissue would further increase the granulation tissue formation.

Patients diagnosed with GPA are generally treated with cyclophosphamide and glucocorticoids per general guidelines for ANCA-related small vessel vasculitis (antibody-associated vasculitis (AAV)) diseases [12]. This initial stage of treatment is often modified based on the severity of GPA progression but can typically induce remission within three to six months, depending on individual patient history and response to treatment. When treated early enough, many patients can manage their symptoms but require continued treatment and monitoring. Without treatment, patients with severe GPA can have a mortality rate of over 90%. However, specialized treatment has been shown to mitigate this prognosis to a five-year survival rate ranging from 74% to 79% for severe cases [13]. Relapse is relatively common, even for patients adhering to treatment plans for GPA, with an incidence rate of 30-60% [14,15]. However, a number of patients do experience complete remission.

## Conclusions

This report illustrates a rare presentation of GPA symptoms and our treatment process from diagnosis to recovery. It is essential to recognize these rare symptoms and to approach this condition in a holistic and comprehensive manner to develop an early diagnosis and provide effective treatment. The complexity of GPA further strengthens the need for this approach, as GPA is a rare condition with multiple presenting symptoms in the head and neck and is a condition that is often lethal if left untreated. Middle ear manifestation of GPA with facial paralysis is an unusual symptom but should not preclude a GPA diagnosis. Instead, otitis media that fails to resolve with traditional treatment and has other systemic symptoms, as detailed in our patient, should raise suspicions about GPA.

Given that lower motor facial palsy is not always related to Bell's palsy, other causes for facial paralysis should be ruled out by compiling comprehensive data consisting of a patient's history, physical examination, serological testing, and imaging studies. The treatment steps outlined in this report, including high-dose oral steroids and Rituximab, may provide crucial information on ways to mitigate symptoms in patients exhibiting these symptoms. The patient's recovery process serves as a testament to the efficacy of this treatment plan. Given the rare incidence of this condition, future studies involving individuals experiencing these rare symptoms will direct our treatment approach to improve health outcomes for GPA management.
